# Long-term telemetric intracranial pressure monitoring for diagnosis and therapy optimisation of idiopathic intracranial hypertension

**DOI:** 10.1186/s12883-021-02349-8

**Published:** 2021-09-07

**Authors:** Victor F. Velazquez Sanchez, Giath Al Dayri, Christoph A. Tschan

**Affiliations:** Department of Neurosurgery and Spine Centre in Ludmillenstift Hospital in Meppen, Ludmillenstrasse 4-6, 49716 Meppen, Germany

**Keywords:** Idiopathic intracranial hypertension, Intracranial pressure monitoring, Telemetric intracranial pressure monitoring, Home-monitoring of ICP, Home-Telemonitoring of ICP

## Abstract

**Background:**

Idiopathic intracranial hypertension (IIH) is a disease which is difficult to diagnose and moreover difficult to treat. We developed a strategy for long-term telemonitoring of intracranial pressure (ICP), by incorporation of the NEUROVENT®-P-tel System, with the goal of improved diagnosis and consequent therapy of this disease. We highlight the results obtained through this approach.

**Methods:**

Twenty patients with suspected IIH who were treated in our hospital from August 2014 to October 2020 (16 females, 4 males, median age 36,6 years), were assigned to one of two ICP monitoring settings, “Home-Telemonitoring” (*n* = 12) and “Home-Monitoring” (*n* = 8). The ICP data were analysed and used conjointly with the accompanying clinical picture for establishment of IIH diagnosis, and telemonitoring was resumed for therapy optimisation of confirmed cases.

**Results:**

The diagnosis of IIH was confirmed in 18 of the 20 patients. Various surgical/interventional treatments were applied to the confirmed cases, including ventriculoperitoneal (VP) shunting (*n* = 15), stenting of the transvers venous sinus (*n* = 1), endoscopic third ventriculostomy (ETV) (n = 1), and ETV in combination with endoscopic laser-based coagulation of the choroid Plexus (*n* = 1). Optimal adjustment of the implanted shunt valves was achieved with an average valve opening pressure of 6,3 ± 2,17 cm H_2_O for differential valves, and of 29,8 ± 3,94 cm H_2_O for gravitational valves. The Home-Telemonitoring setting reduced consequent outpatient visits, compared to the Home-Monitoring setting, with an average of 3,1 visits and 4,3 visits, respectively. No complications were associated with the surgical implantation of the P-tel catheter.

**Conclusion:**

This study offers insight into the use of long-term ICP monitoring for management of IIH patients in combination with dual-valve VP shunts. The use of NEUROVENT**®** P-tel system and potentially other similar fully implantable ICP-monitoring devices, albeit invasive, may be justified in this complex disease. The data suggest recommending an initial adjustment of dual-valve VP-shunts of 30 and 6 cm H_2_O, for gravitational and differential valves, respectively. Further research is warranted to explore potential integration of this concept in IIH management guidelines.

## Background

Idiopathic intracranial hypertension (IIH) is a disorder of unknown etiology, characterized by increased intracranial pressure (ICP) without radiological evidence of intracranial pathology except for occasional empty sella turcica, distension of the optic nerve sheath, flattening of the posterior globes of the eye, and potential stenosis or collapse of the transverse venous sinus [6, 9, 12, 18].

Some authors suggest the use of the term “secondary intracranial hypertension” if the etiology of the elevated ICP was clear, for instance as a side effect of medical treatment, or as part of post-thrombotic transverse sinus stenosis; however, it is unclear if sinus stenosis in association with IIH is a primary cause or a consequence of the elevated ICP [9, 20]. The term “pseudotumor cerebri” (which translates from Greek and Latin to false tumour of the brain) is widely used as an alternative to IIH, arising from the notion that the clinical findings in these patients are similar to having a brain tumour without actually having one. The term “benign idiopathic intracranial hypertension” has also been used as far as the 1950s; however, many scholars including the authors of this paper, find the use of the term “benign” inappropriate, due to the causal association of this disease with potential visual loss [18].

IIH typically affects obese women in childbearing age, rendering female sex and obesity risk factors [5], and the incidence of this disease over the years has increased, with correlated increase in the prevalence of obesity in all ages and genders [12, 18]; however, patients with other demographic profiles have also proven to be affected by it [6, 9, 12, 18], adding to the difficulty of establishing IIH diagnosis.

The characteristic chief complaint is a bilateral frontal or retro orbital headache. Visual and auditory symptoms such as transient visual obscuration and pulsatile intracranial noises are also common, with some patients additionally complaining of back and neck pain, dizziness, photophobia, nocturia, diplopia, cognitive impairment, and radicular pain [18]. Many factors render the diagnosis of this disease difficult, such as low disease incidence (estimated to be around 4.7 per 100.000 in the general population [13]), the unspecific signs and symptoms associated with this disease, and lack of clear radiological findings especially in the early phases of this disease, combined with the lack of reliable non-invasive methods for measurement of the ICP for confirmation of intracranial hypertension; which is why some patients may suffer for years before diagnosis of IIH is established. IIH patients are stigmatized as difficult-to-treat patients, and since the chief complaint in most cases is headache, which is subjective, it is frequently not addressed as promptly as it should. The social aspects associated with IIH-related hindrances due to the frequent symptoms of headache and vision loss may also be overlooked, leading to negative patient and society outcome, which is why it is important to address these aspects, and study them furtherly.

A set of criteria was described by Dandy in 1937 (Dandy Criteria), followed by development of the Modified Dandy Criteria (Table 1), which are currently used to various extents in the diagnosis of IIH [18].

**Table 1:** Modified Dandy criteria for IIH used in the IIHTT (reproduced from Wall 2017 [24]).

1	Signs and symptoms of increased ICP
**2**	Absence of localizing findings on neurologic examination
**3**	Absence of deformity, displacement, or obstruction of the ventricular system and otherwise normal neurodiagnostic studies, except for increased cerebrospinal fluid pressure (> 200 mm H_2_O). Abnormal neuroimaging except for empty sella turcica, optic nerve sheath with filled out CSF space, and smooth-walled non flow-related venous sinus stenosis or collapse should lead to another diagnosis
**4**	Awake and alert
**5**	No other cause of increased ICP present

The guidelines of the German Society of Neurology recommend careful documentation of the medical history of the patient, and exclusion of other factors that may raise the ICP, thus mandating thorough neurological and ophthalmological examinations. Lumbar punctures have been used extensively in the diagnosis of IIH, through pressure measurement and CSF analysis, but also through positive patient responses by alleviation of symptoms, such as headaches. CT or MRI scans with venography sequences or digital subtraction venography are also recommended for diagnosis of venous sinus stenosis, which occasionally correlates with IIH [9].

ICP measurement, which is crucial in the diagnosis of IIH, can be conducted in various methods, and these can be classified into invasive and non-invasive methods. The invasive methods measure ICP directly in one of the intracranial spaces, namely epidural, subdural, intraparenchymal, or intraventricular spaces [14]. Non-invasive indirect methods for measurement of ICP or identification of intracranial hypertension have also been developed; however, they are not reliable enough to be used on regular basis. Upon diagnosis, the German Society of Neurology identifies three stages under which the IIH patient is classified and accordingly treated; these are highlighted in Table 2.

**Table 2:** Clinical stages of IIH and their corresponding recommended treatment. (Translated and reproduced from the website of the German Society for Neurology – DGN. https://dgn.org [24])

Clinical Stage	Corresponding Recommended Treatment Strategy		
**Stage I:** IIH without focal neurological deficits (mild papilledema)	• Weight reduction • Acetazolamide (with regular control of blood Potassium level) • Alternatively: Topiramat + Furosemide (with regular control of blood Potassium level)		
**Stage II:** IIH with: • Significant papilledema and/or • Sight impairment or visual field loss	• Treatment for Stage I with: • Successive LPs (nearly every 2 weeks) until pressure is < 20 cm H_2_O	Goals: • Reduction of Pressure < 18 cm H2O And	Additional Measures:S • Reduction of acetazolamide Dosage And
**Stage III:** IIH with: • Progressive loss of sight and/or • Rapid progressive onset of sight impairment or Visual field loss.	• Treatment for Stage I and II with: • Neuroradiological Intervention (stenting) and/or • Surgical Intervention o CSF diversion (VP, lumbo-peritoneal Shunting) o Optic nerve sheath fenestration (ONSF)	• Normalising of sight	• Regular Control of CSF opening pressure and clinical symptoms

Medical treatment was well studied in the Idiopathic Intracranial Hypertension Treatment Trial (IIHTT), in which statistically significant improvements in visual function, quality of life measures, papilledema grade, and CSF pressure were achieved using acetazolamide [6]; other related medications include Topiramat and Furosemide. Neurologists often integrate repeated lumbar punctures every few days, which albeit tedious, is quite an affective therapeutic option in IIH [20].

Surgically, the most applied technique is CSF diversion, mostly through ventriculoperitoneal (VP), ventriculoarterial (VA), or lumboperitoneal shunts, with shunt revision operations being more frequent in lumboperitoneal shunts than in VP shunting [9]. Currently, VP and VA Shunting using systems that combine differential and gravitational valves in complex IIH cases constitute the recommended CSF shunting approach [8]. Endoscopic ventricular surgery solutions such as ETV and choroid plexus coagulation are being increasingly deployed as an alternative or in combination with other surgical treatments. Elevated ICP may correlate with venous sinus stenosis, and although the physiopathology is unclear [6, 18], interventional treatment is also possible via venous sinus stenting in case of stenosis thereof, which requires life-long use of Antiplatelet drug therapy.

Optic nerve sheath fenestration (ONSF) is a surgical option to decompress the optic nerve sheath in cases of severe sight impairment [20]. With obesity being a considerable risk factor in IIH, surgery for weight reduction has been successful in treating IIH and reducing associated papilledema as shown by Fridley et al. [16]

Although there is a wide variety of diagnostic and therapeutic options for IIH, some of which have been successful in limiting progressive vision loss, they still struggle in terms of providing sufficient alleviation of other symptoms of this disease, mainly because the IIH patients tend to be overly sensitive to the slightest VP shunt adjustment, exaggerating problems of over- and underdrainage of the shunt system, and leading to significant headache, which is often difficult to correctly attribute to over- or underflow, adding to the complexity of disease management. Another problem is the complexity of the new shunt devices with additional pressure/flow settings, which is combined with the interpatient variability of ICP at which symptoms are developed. All these issues warrant the need for long term and extensive ICP monitoring, before and after therapy, for optimisation of the patient outcome through individualised therapy and follow-up, rather than one-for-all solutions.

However, to our knowledge, there are no published clinical trials that have incorporated long-term ICP monitoring exclusively in IIH patients. In this respect, the NEUROVENT®-P-tel catheter was developed for long-term ICP-monitoring in complex cases related to hydrocephalus, and first clinical results of this device were published less than a decade ago [19]. It is superior to other simple approaches, such as lumbar puncture, in terms of its ability to provide long-term ICP monitoring, and it offers higher correlation of ICP values to the usual activities of the patient compared to the hospital [15], and potentially a lower risk of infection compared to open systems with continuous cable connection.

Based on this concept, we established a new strategy for optimised diagnosis and subsequent treatment of IIH, by incorporation of telemetric ICP monitoring in these processes using NEUROVENT®-P-tel catheter. In this study, we highlight the results obtained through this approach, and reflect on them in terms of safety, efficiency, reproducibility, shortcomings, potential improvements, and relevance on overall outcome of IIH patients treated through it.

## Methods

In the period between August 2014 and October 2020 the authors applied a workflow plan for the management of patients with diagnosed or suspected IIH who were presented at the Neurosurgery Department in Ludmillenstift Hospital in Meppen, Germany (Figure 6). An integral part of this workflow was the surgical implantation of a device that allows for long-term monitoring of ICP in these patients while they are at home, followed by analysis and utilisation of the extracted data for the diagnosis and follow up of these patients, as well as the application of the ICP findings in combination with the correlated symptoms for further therapy modifications after surgeries, for instance setting-optimisation of adjustable shunt-valves. From a total of 78 hospital admissions with suspected IIH, the workflow structure was applied to all patients who were willing to participate in the surgery required for its implementation, ending up with 20 patients (16 females, 4 males, median age 36,6 years). These patients were assigned to one of two ICP monitoring settings which are discussed below, being “Home-Telemonitoring” (*n* = 12) and “Home-Monitoring” (*n* = 8).

### The NEUROVENT® telemetric ICP measurement system

The ICP monitoring system NEUROVENT® P-tel (Raumedic AG, Helmbrechts - Germany) has been extensively discussed before [2–4, 19]. It is certified for use in Europe, and it was the first CE-certified device for telemetric ICP monitoring [10], with a standard implantation period of up to 3 months; nevertheless, an extension of the period of implantation beyond 3 months may be granted if the patient signed a special exemption form in which he or she are made aware of the standard implantation period and potential risks of prolonged implantation. This arrangement was presented before a special ethics committee, which approved to it.

**Fig. 1:**
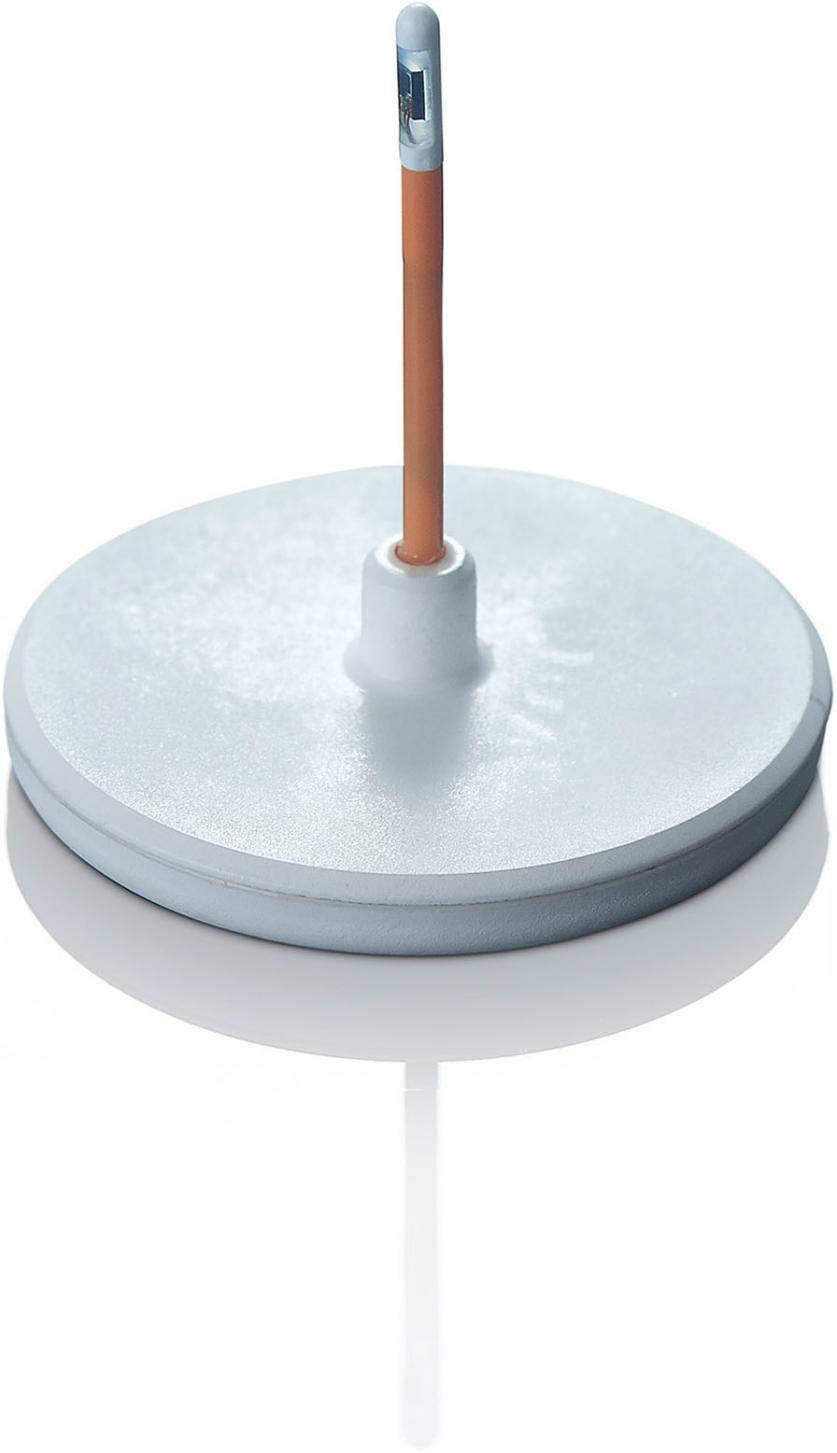
The implantable P-tel unit. (Copyrighted image obtained from©Raumedic AG, written permission was granted for its use in this publication)

The system consists of two integral units, the first is a below-the-scalp fully implantable unit (the NEUROVENT P-tel, Figure 1), consisting of a Silicon membrane that serves as a piezoresistive pressure transducer, and a microchip in a ceramic housing for data processing.

**Fig. 2:**
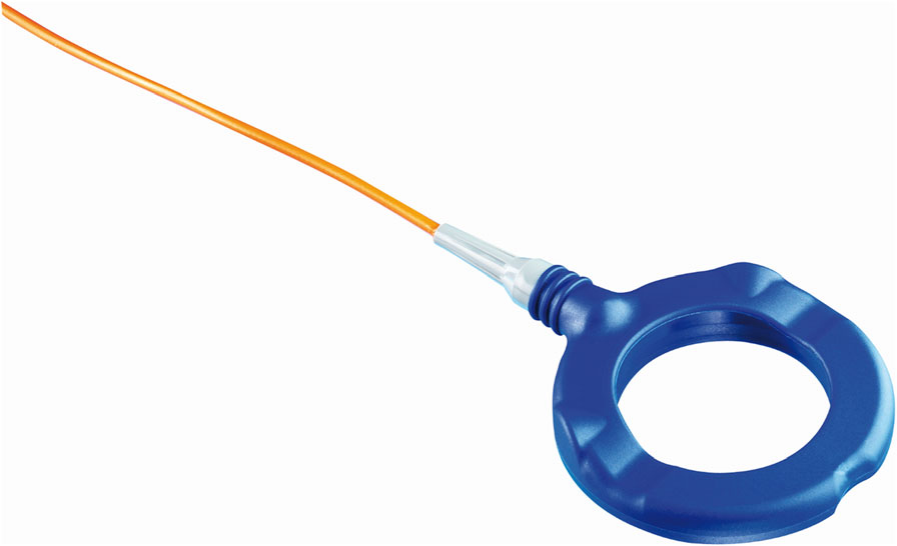
The Antenna/Reader unit labelled TDT1 readP. (Copyrighted image obtained from©Raumedic AG, written permission was granted for its use in this publication)

**Fig. 3:**
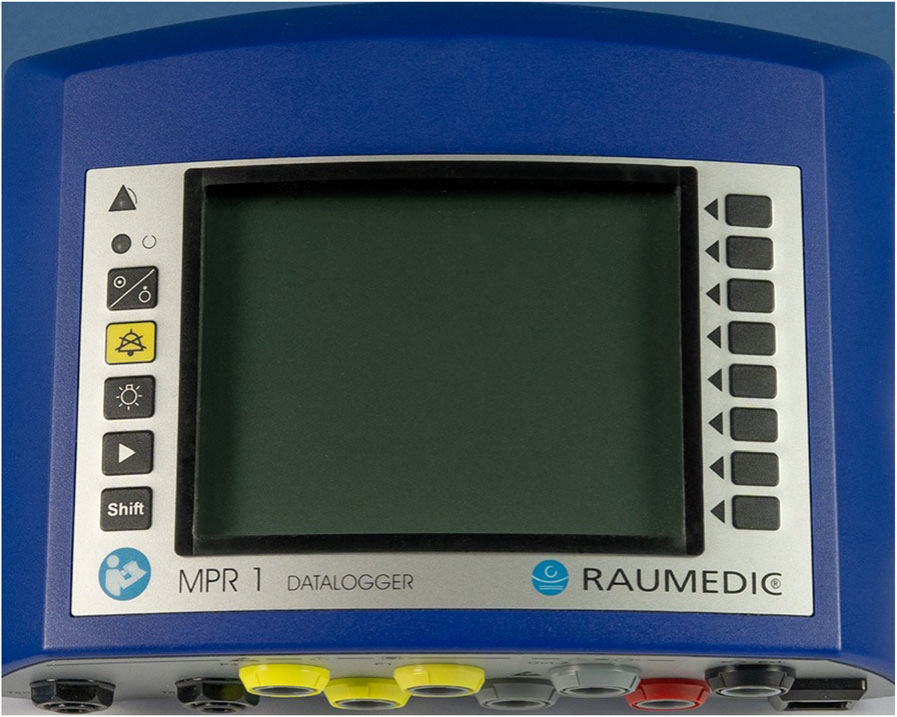
The interactive display/storage unit or DATALOGGER. (Copyrighted image obtained from©Raumedic AG, written permission was granted for its use in this publication)

The other part is the external unit, consisting of an antenna labelled “Reader TDT1 readP” (Figure 2), which is applied to the head skin, and connected to an interactive display/storage unit labelled “MRP 1 DATALOGGER” (Figure 3).

**Fig. 4:**
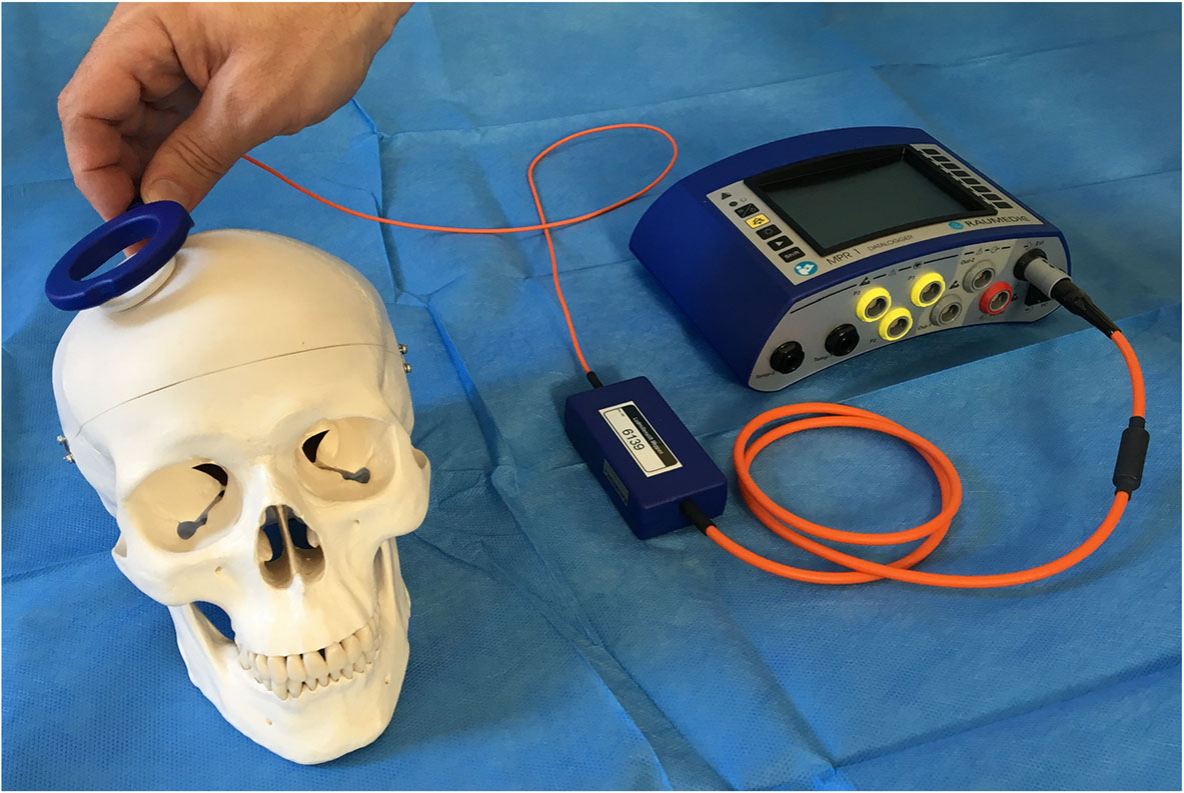
Illustrating the NEUROVENT® ICP-Monitoring System and its approximate relation to the skull during ICP monitoring. The P-tel catheter is usually implanted in the left or right frontal lobe below the scalp, and the circular reader is applied on the skin just above the palpable P-tel catheter and fixed with a bandage or adhesive tape for the measurement to take place. (Photo is taken and copyrighted by the authors of this publication)

The implanted catheter records and processes ICP measurement at a frequency of 1 or 5 Hz, and the reader/antenna is applied to the scalp in the vicinity of the implanted P-tel unit to capture the pressure measurement values transdermally from it, through electromagnetic induction, furtherly transmitting these values to the DATALOGGER, allowing the measurements to be stored and displayed on the screen, as well as allowing control over the settings of the device. The external unit with both the reader and datalogger parts and its relation to the patient’s skull and catheter during ICP monitoring are illustrated in Figure 4.

### Home-monitoring and home-Telemonitoring of ICP

Home-Monitoring and Home-Telemonitoring are two comparable settings for long-term ICP monitoring using the NEUROVENT® P-tel which are well described by Tschan et al. [17]. In both settings, the P-tel catheter is implanted surgically through a burr hole which is typically located in the frontal lobe of the non-dominant hemisphere. (The exact methodology of implantation has been described extensively in multiple publications [2, 17, 19]). Afterwards, following an initial measurement in the clinic, the patients or legal guardians in case of children are taught to manage the NEUROVENT® P-tel device, before being discharge from the hospital.

The difference between both Home-Monitoring and Home-Telemonitoring settings mainly lies in the frequency at which the patient must be presented in the outpatient clinic for reading the device. The reason for requiring two monitoring settings is related to the limited storage capacity of the MPR Datalogger unit, which has a storage capacity of 72 h of continuous ICP measurement at 5 Hz (i.e. 5 ICP measurements per second) mode and 14 days at 1 Hz mode. Therefore, in the **Home-Monitoring setting**, when the device storage is full (after a period between 3 and 14 days of continuous ICP monitoring, depending on the measurement frequency), the patient must come to the outpatient clinic so that the data are transferred and/or analysed by the attending physician, and the old data in the Datalogger are then deleted, creating more storage space for another cycle of ICP monitoring [17]. Whereas in the **Home-Telemonitoring setting**, an alternative approach is taken to alleviate the need for frequent outpatient visits, and this is achieved by lending the patients a portable computer with pre-installed software (**Raumedic DataViewer** and **TeamViewer**), and they are taught to use the software at home, so that when the device storage is at full capacity, they transfer the data to the given portable computer using Raumedic DataViewer software, which restores the capacity of the ICP monitoring device, enabling further ICP monitoring. Additionally, TeamViewer, which is a password-protected screen-sharing and remote-control software that integrates the technology of virtual private network (VPN), was used in teleconferences between the patient and physician, allowing the attending physician to have an overview of the ICP data and analyse them remotely.

Between Home-Monitoring and Home-Telemonitoring, the decision of assigning patients to one setting or the other depended on a few factors, ranging between suggestive and exclusionary. Patients who lived near the outpatient clinic were mostly managed in the Home-Monitoring setting. Patients who did not possess basic computer literacy or internet access at home were excluded from the Home-Telemonitoring setting, and were managed in the Home-Monitoring setting, as the alterative setting that requires active patient involvement in dealing with data analysis was not feasible. Patients living far away from the outpatient clinic were optimally managed in the Home-Telemonitoring setting whenever possible.

After the ICP monitoring data have been analysed, medical decisions were made, and patients who underwent surgery mostly retained their implanted P-tel devices, and ICP monitoring was resumed for the purpose of follow-up and potential optimisation of the shunt-valve setting after shunt operations. After the management has been concluded according to the workflow plan, the NEUROVENT**®** P-tel catheter was explanted.

### Data analysis of telemetric ICP measurements

For analysing the telemetric ICP measured data, we established a documentation sheet using the Raumedic DataView software. ICP data were analysed not only in terms of their absolute values, but they were also analysed for ICP curves over time, ICP curve amplitudes, and the presence of the pathologic Lundberg ICP waves A, B or C. In this regard, details of analysing telemetrically obtained ICP data in a standardised approach have been described before [7, 11, 17].

Patients also documented their activities and symptoms on daily basis, supplying the physician with additional information regarding the position in which the analysed ICP was measured, together with ICP values at which symptoms such as headache or nausea took place.

In the cases where monitoring occurred in a Home-Monitoring setting, data analysis occurred in the outpatient clinic. The patient had to present himself or herself in the outpatient clinic to download ICP data from the MPR Datalogger for gaining new storage capacity on the device and/or data analysis, upon which further treatment decisions were made. In cases in which Home-Telemonitoring took place, patients were given an appointment for a teleconference after they were discharged from the hospital. In this teleconference, the attending physician is granted remote access by the patient to have an overview of the ICP data, which is conducted in a VPN setting to ensure the privacy of the patient. If ICP data had to be analysed on the computer of the attending physician, they were sent anonymously, in the form of numerical sequences, to emphasize privacy.

**Fig. 5:**
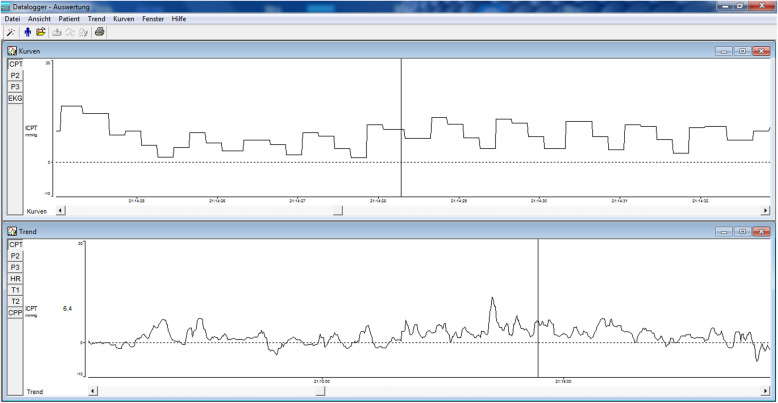
Remote analysis of ICP measurement curves of a patient with suspected IIH, utilizing TeamViewer software. Screenshot from the physician’s PC. (Photo is taken and copyrighted by the authors of this publication)

In both settings, an evaluation sheet which is based on the ICP values was filled by the attending physician for the purpose of documentation of tangible findings and assisting in further decision making. These monitoring sessions were either repeated or ended, potentially being followed by an active treatment, and concluding with further ICP monitoring sessions for follow-up and treatment optimisation. Figure 5 shows a screenshot of the physician’s computer using the TeamViewer software in a Teleconference.

### Patient profiles

Telemetric ICP monitoring was applied in 20 patients with suspected or diagnosed IIH, who were presented at our neurosurgery department, during the period between August 2014 and October 2020, and were willing to participate in this workflow structure. Patient data are summarized in Table 3.

**Table 3:** General data of all 20 patients enrolled in this study

ID	Sex	Main Symptoms	Papilledema	Previous Treatment	Type of Follow Up	Distance between home and clinic in kilometres
1	F	Headache	No	Topiramat	Home-monitoring	21
2	F	Headache	No	None	Home-monitoring	29
3	F	Sight impairment	Yes	Acetazolamide, Topiramat, furosemide, weight loss	Home-Tele-monitoring	174
4	F	Headache, sight impairment, dizziness	No	Acetazolamide, LPs	Home-monitoring	45
5	M	Headache, sight impairment	Yes	Acetazolamide, LPs	Home-Tele-monitoring	559
6	F	Headache, sight impairment	Yes	Acetazolamide, LPs	Home-Tele-monitoring	45
7	F	Headache	No	Acetazolamide, Topiramat	Home-Tele-monitoring	586
8	F	Dizziness	No	VP Shunt Codman valves	Home-Tele-monitoring	137
9	M	Headache, sight impairment	Yes	LPs	Home-Tele-monitoring	45
10	F	Headache, sight impairment	No	Acetazolamide, LPs	Home-monitoring	109
11	F	Headaches	No	LPs	Home-monitoring	21
12	F	Headaches	No	None	Home-Tele-monitoring	58
13	F	Headache, sight impairment (Blind)	No	None	Home-Tele-monitoring	740
14	F	Headache,	No	VP Shunt ProGAV valve with Shunt-Assistant (20 cm H2O)	Home-Tele-monitoring	158
15	M	Headache, nausea, vomiting	Yes	None	Home-monitoring	42
16	M	Headache, sight impairment	Yes	LPs	Home-monitoring	5
17	F	Headaches	No	Sumatriptan, Topiramat	Home-Tele-monitoring	178
18	F	Headaches	No	VP Shunt	Home-Tele-monitoring	190
19	F	Headaches	Yes	None	Home-monitoring	42
20	F	Headaches	No	Acetazolamide, LPs	Home-Tele-monitoring	112

The age of these patients ranged from 11 to 58 years, with an average of 36, 65 years. Four patients where younger than 18 years and the sex ratio was 4 males to 16 females. The chief complaint of these patients was headache. In 15 cases, previous therapy was already initiated, mostly with acetazolamide or a combination of acetazolamide and weekly lumbar punctures in accordance with the recommendations of the German Society for Neurology, with additional VP shunt surgery in 3 out of these 15 previously treated patients. In the other 5 cases, the patients were not subjected to previous treatments. In terms of additional clinical manifestations, 7 out of the 18 diagnosed IIH patients were presented papilledema before the implantation of the P-tel catheter.

In accordance with inclusionary and exclusionary criteria which were discussed earlier in this article, 12 patients were manged in a Home-Telemonitoring setting, while the other 8 patients were enrolled under the Home-Monitoring setting. In the cases where Home-Telemonitoring was done, distance between the patient’s home and the clinic was on average 248,5 Km (min. 45 km and max. 740 km), whereas as the average distance to the clinic in Home-Monitoring setting was 39,25 Km (min. 5 km and max. 109 km).

Most of the IIH patients in our cohort were treated by VP-shunting, using the most advanced available shunt systems that combined 2 adjustable valves (classical differential valves, and gravitational valves with opening pressure that is dependent on the posture of the patient, to minimise over-drainage). The shunt systems used were either the proSA® Shunt System (with proSA® gravitational and proGAV® differential valves), or the newest generation M.blue plus® shunt system (with M.bleu gravitational and proGAV differential valves), both manufactured by Miethke GmbH, Germany.

### Workflow plan

**Fig. 6:**
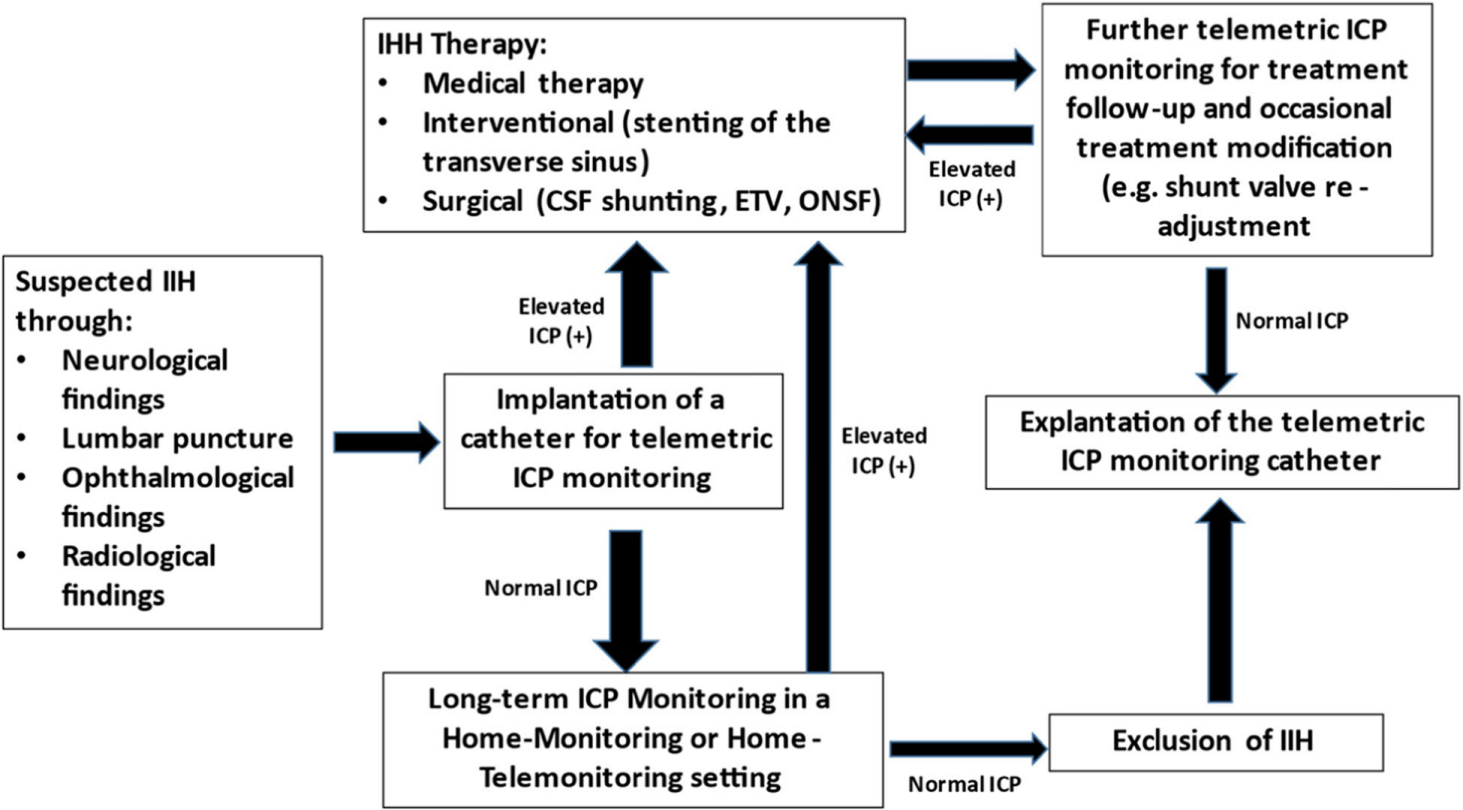
Workflow plan for management of IIH patients which integrates telemetric ICP monitoring

In this series of patients, the ICP catheter was implanted in patients with suspected IIH or in cases where treatment of IIH was already initiated but not satisfactory. An initial ICP monitoring over the next few days postoperatively was conducted in hospital. A combination of clinical, radiological, and ICP parameters (typically above 15 mmHg at symptomatic episodes) were assessed, for the initial diagnosis of IIH to be established, and treatment was then initiated for these patients. The patients were discharged from the hospital when all necessary diagnostic and therapeutic measures were applied, including patient training for dealing with necessary hardware and software elements of the telemetric ICP device. Patients in which IIH was suspected but did not fit the typical criteria also maintained the implanted P-tel catheter for further long-term ICP monitoring. The long-term telemetric ICP monitoring phase was then initiated within the Home-Monitoring or Home-Telemonitoring settings for follow-up and further evaluation. The goal of subsequent adjustments of the shunt valves was mainly to address persistent symptoms like headache, and the long-term ICP monitoring assisted in deciding the direction and amplitude of adjustment, through identification of symptomatic overflow or underflow of CSF through the implanted VP shunts. See Figure 6 for a summary of the management workflow plan.

## Results

The combined duration in which the P-tel catheter was implanted in all patients in this cohort was 4891 days, with a median of 129.5 days per patient (min. 17 days and max. 1554 days), of which, 7763 h of combined ICP measurements were recorded, yielding an average of 388 h of ICP measurement per patient. Table 4 shows the total and mean measuring times of the entire cohort both at hospital and home.

**Table 4:** ICP Measuring Times in the hospital and at home

	Total ICP- Monitoring Time	ICP Measured at Hospital	ICP Measured at Home
**Sum**	7763 h	2297 h	5466 h
**Mean**	388,15 ± 192,08 h	114,85 ± 62,62 h	273,3 ± 209,76 h
**IQ Range**	229–562 h	62–146,5 h	73–483 h

In the period in which the 12 patients who were followed-up through the Home-Telemonitoring setting, 83 teleconferences occurred, averaging 6 sessions per patient (6 ± 2,23, min. 4 and max. 13); and in the follow-up period, these patients were presented in the outpatient clinic 3,16 times on average (3,16 ± 1,86, min. 0 and max. 8). Patients who were followed-up by Home-Monitoring presented in the outpatient clinic 4,37 times on average (4,37 ± 2,81, min. 1 and max. 10). The individual recording time for patients from both monitoring settings as well as the number of conducted teleconferences are listed in Table 5.

**Table 5:** Individual recording times of patients enrolled in the Home-Monitoring and Home-Telemonitoring settings

Home-Monitoring				Home-Telemonitoring				
	Measured Time in Hours				Measured Time in Hours			
**ID**	**In Hospital**	**At home**	**Number of Out-Patient Visits**	**ID**	**In Hospital**	**At Home**	**Number of Out-Patient Visits**	**Number of Teleconferences (Home-Telemonitoring only)**
**1**	49	3	1	**3**	100	677	1	5
**2**	136	125	3	**5**	79	483	2	7
**4**	24	282	3	**6**	80	67	8	10
**10**	326	51	3	**7**	77	536	4	10
**11**	115	30	2	**8**	61	756	5	5
**15**	157	161	3	**9**	100	677	2	4
**16**	136	125	10	**12**	63	390	5	13
**19**	25	175	10	**13**	79	483	0	4
				**14**	15	79	2	6
				**17**	233	26	3	5
				**18**	177	140	1	6
				**20**	265	200	5	8

### Therapeutic consequences of ICP recordings in IIH

After the P-tel catheter was implanted and diagnostic measurements were conducted, IIH was confirmed in 18 of 20 patients. After exploration of accessible therapeutic options and extensive discussion with the patient, individual therapy was initiated, and the choice of applied treatment was influenced by the preference of the patient. Most patients were managed by the implantation of VP shunts with differential and gravitational adjustable valves. In other cases, endoscopic treatment with laser-based choroid plexus coagulation and/or ETV and was applied instead. One patient underwent radiological stenting of the transvers sinus as a primary treatment.

In 2 patients, long-term telemetric ICP monitoring lead to the exclusion of IIH. In one case, this was concluded at an early stage of the diagnosis, so no therapy was initiated previously or afterwards. In the other case, the patient was already treated as IIH with VP shunting at presentation, so after the ICP measurement in a Home-Telemonitoring setting dismissed the diagnosis of IIH, the VP shunt was surgically removed. Table 6 lists the definitive applied therapy in this cohort, together with the therapeutic settings of the adjustable shunt valves and number of subsequent adjustments postoperatively.

**Table 6:** Definitive therapeutic choice and shunt settings in patients with diagnosed IIH after conducting telemetric ICP monitoring

ID	The Definitive Applied Therapy	VP-shunt valve pressure settings in cm H_2_O (differential/gravitational) on conclusion of ICP monitoring*	Number of subsequent shunt valve adjustments after surgery
1	VP Shunt (M.blue plus®)	5/25	0
2	ETV + Plexus chorideus coagulation	Not Applicable	0
3	Stenting of the transverse sinus	Not Applicable	0
4	VP Shunt (proSA® Shunt System)	5/28	2
5	VP Shunt (proSA® Shunt System)	7/30	2
6	VP Shunt (proSA® Shunt System)	0/36	6
7	VP Shunt (proSA® Shunt System)	10/24	4
8	Change of VP Shunt valves to proSA® Shunt System	6/26	3
9	VP Shunt (proSA® Shunt System)	8/30	3
10	VP Shunt (proSA® Shunt System)	6/30	3
11	VP Shunt (M.blue plus®)	10/34	2
12	VP Shunt (proSA® Shunt System)	9/36	5
13	VP Shunt (proSA® Shunt System)	8/28	0
14	Explantation of VP Shunt. Exclusion of IIH.	Not Applicable	2
15	Exclusion of IIH; no treatment for IIH	Not Applicable	0
16	VP Shunt (proSA® Shunt System)	8/20	3
17	ETV	Not Applicable	0
18	Changes of VP Valves to proSA® Shunt System	7/32	2
19	VP Shunt (proSA® Shunt System)	2/40	6
20	VP Shunt (proSA® Shunt System)	4/28	5
**Average therapeutic shunt valve setting in the cohort**		**6,3/29,8**	**Total number of adjustments in all patients = 48**
**Average therapeutic shunt valve setting excluding patients under 18 years of age**		**6/30,58**	

*The 2 numbers separated by a slash refer to the valve settings of the differential and gravitational units, respectively. The utilised shunt systems were ProSA® Shunt System (Miethke GmbH, Germany) and M.blue plus® (Miethke GmbH, Germany)

### Shunt valve adjustment and alternative therapy

Home-Monitoring and Home-Telemonitoring were utilised for treatment optimisation after shunt operations. Subsequent VP valve adjustment after surgeries was applied using system-compatible magnetic-based instruments, and it took place for a total of 48 times in this cohort. Valve readjustment took place for modification of CSF flowrate, and the amplitude of change, as well as direction (more or less flow) and choice of valve to adjust (differential, gravitational, or both) were dependant on the objective ICP values during monitoring, as well as the associated subjective patient symptoms. At the end of the treatment optimisation period we found in this cohort that patients with IIH who were treated with CSF shunting to have an average differential valve setting (opening pressure) of 6,3 ± 2,17 cm H_2_O, and gravitational valve setting of 29,8 ± 3,94 cm H_2_O; By excluding patients who were under 18 years, these average values were still comparable, at 6 ± 2,3 cm H_2_O and 30,58 ± 4,18 cm H_2_O for differential and gravitational valves, respectively. Table 6 highlights the respective shunt valve settings in these patients.

In both cases where endoscopic measures were performed (ETV with and without laser-based coagulation of the choroid plexus), they were shown to be sufficient treatment in the follow up period. Similarly, the patient who underwent stenting of the transverse sinus did not require further treatments.

### Complications

In this patient series, no major complications occurred with the insertion and maintenance of the telemetric probe. In this regard, no incidents of seizures, wound healing disturbances, haemorrhage, or intracranial infections occurred. Moreover, no emergencies took place during home telemonitoring, for instance in association with elevated ICP. In three patients, the external MPR DATALOGGER had to be changed due to malfunction after being accidentally dropped on the ground. No difficulties occurred at home in the management of the corresponding Home-Telemonitoring software.

## Discussion

The P-tel system offered a source of objectiveness in the management of IIH. Many authors describe this tool as a secure and reliable method for obtaining long-term ICP measurements with a low complication rate [1]; this was in correlation with the findings in this study, which reported no major complications related to the implantation of the P-tel catheter in all 20 patients. However, it is an invasive surgical procedure which must be repeated at least once, for the removal of the catheter. And albeit relatively safe, this approach carries the potential of complications, such as intracranial bleedings and infections. The lack of alternative reliable long-term ICP monitoring approaches as described by Zhang et al. [21] allows for this risk to be tolerated; however, the risk of surgery must be weighed against alternative measures in the management of each patient, before deciding for this approach.

The average implantation periods were highlighted in the results section, with the maximum being 1554 implantation days in one patient before surgical removal, without demonstrating any related complications; however, due to the relatively small sample size of this cohort, these results are not enough to make conclusions regarding the long-term safety of the P-tel catheter; and this creates a research opportunity which is based on combining data from this cohort as well as other similar studies in a systemic review, for the purpose of determination of long term safety beyond 3 months of implantation, and potential consequent modification of the recommended implantation guidelines.

Assigning the patients to the Home-monitoring or Home-Telemonitoring settings could not be randomised, as the two settings were developed to address inherent objective differences between patients, such as patient proximity to the clinic, internet availability, and computer know-how; and the decision also took the patient’s preference in consideration.

Home-Telemonitoring provided larger amounts of data of long-term high frequency ICP monitoring without interruption, which was not possible beyond 3 days in the Home-Monitoring setting, due to the limited storage capacity of the Datalogger. In this regard, we argue that with the exponential growth of digital storage devices, it is quite tangible to apply design modifications of a device comparable in size to the Datalogger, for the purpose additional storage, and potentially higher measurement frequency beyond 5 Hz to accommodate to pulse-pressure variations, thus reducing the necessity for frequent presentation in the outpatient clinic solely for data transfer and freeing storage space on the device. The larger amount of data produced in the Home-Telemonitoring setting was useful, but this setting required extensive training of the patient for management of the software and hardware aspects of the P-tel system, and it was also more demanding and time consuming for the physician, requiring him to be available at teleconferences, sometimes after work hours, and it necessitated additional effort for data analysis.

In case of utilising telemetric ICP monitoring for valve adjustment in IIH patients who were operated with a shunt (e.g. VP shunt), the patient must be presented at the outpatient clinic for manual readjustment, and telemetric ICP monitoring is then resumed at home. In this regard, potential research opportunities lie in the development of digitally - and thus remotely - adjustable shunt valves, furtherly reducing the necessary frequency at which the patient is presented in the outpatient clinic. Patients treated with VP-shunts in this cohort were assigned modern shunt systems with combined differential and gravitational adjustable valves (as opposed to shunt systems with non-adjustable gravitational valves or differential valves only); this provided additional fine tuning and a better chance of reaching a satisfactory therapeutical valve setting, through the additional adjustable gravitational unit that minimises postural over-drainage of shunts. The results of this study also gave an important insight with respect to the average valve setting in IIH patients treated with VP shunts, which was found to be 6,3/29,8 cm H_2_O (differential/gravitational) in the entire cohort, and 6/30,58 cm H_2_O (differential/gravitational) when patients below the age of 18 were excluded. Accordingly, we recommend an initial VP shunt valve adjustment of 6/30 cm H_2_O in adults (differential/gravitational), which statistically minimises the need for consequent valve setting readjustments. A larger number of patients in future similar studies may suggest modification of this recommended initial setting.

An additional significant advantage in the adoption of telemonitoring in IIH was the possibility of attaining real-time objective feedback to the patients themselves, by having an overview of the ICP monitoring, which helped the patients understand the etiology of their symptoms; such as familiarising themselves with ICP values at which the symptoms take place with various activities, and differentiating between headache patterns associated with overflow of VP shunts and those with underflow thereof; however, due to the inherent limitations of a retrospective cohort study, there is no proof that this understanding translated to objective outcome improvement from the patient perspective, compared to the standard management. Nevertheless, it assisted the physician in the process of decision making, for instance in deciding for the direction and amplitude of readjustment of shunt valve settings, potentially reducing the necessary number of subsequent shunt-valve readjustments.

Home-Telemonitoring was used in this cohort for follow-up of interventional stenting for the single patient who was subjected to this therapy, and it was also used for diagnosis and follow-up of patients who underwent endoscopic therapeutic procedures such as ETV and choroid plexus coagulation. And while all the patients in this cohort (*n* = 3) benefited from these approaches which were alternative to shunt implantation, the sample was too small to make conclusions regarding their therapeutic relevance in IIH.

Another interesting area in which a similar design could be deployed is ICP monitoring in association with Acetazolamide therapy, which was not possible in our study, because the majority of patients in this cohort were already treated conservatively by a neurologist, before they were referred to our neurosurgery department for further treatment. Such design adds a potential benefit of objectifying the clinical improvement documented in the IIHTT study, for further understanding of the influence of Acetazolamide and weight reduction approaches (such as gastric banding) on ICP in IIH, but it also warrants close collaboration of various health disciplines such as neurology, neurosurgery, radiology, bariatric surgery, psychology, and nutrition therapy; and we argue that a tangible approach for ensuring such collaboration in IIH is through the modification of the established guidelines related to this disease.

The workflow plan which was incorporated in this study (Figure 6) allowed the integration of many variables in its structure, ranging from inclusion of IIH patients but also those who would ultimately be excluded from this diagnosis, to the integration of various therapy approaches and their varying responses. This structure can be incorporated in future studies that may even slightly differ from this one, and if integration of this technology became more common, and showed consistent improvement upon the current diagnostic and therapeutic standards, it would prompt modification of current IIH management guidelines to include this technology for improved patient outcome.

## Conclusion

This study offers insight into the use of long-term ICP monitoring for management of IIH patients in combination with dual-valve VP shunts. The use of NEUROVENT**®** P-tel system and potentially other similar fully implantable ICP-monitoring devices, albeit invasive, may be justified in this complex disease. The data suggest recommending an initial adjustment of dual-valve VP-shunts of 30 and 6 cm H_2_O, for gravitational and differential valves, respectively. Further research is warranted to explore potential integration of this concept in IIH management guidelines.

## Data Availability

The datasets used and/or analysed during the current study are available from the corresponding author on reasonable request. The raw ICP Data are in RDL-File format that requires special software (RAUMED DataView) to be accessed.

## Abbreviations

CSF: Cerebrospinal Fluid
ETV: Endoscopic Third Ventriculostomy
ICP: Intracranial Pressure
IIH: Idiopathic Intracranial Hypertension
IIHTT: Idiopathic Intracranial Hypertension Treatment Trial
LP(s): Lumbar Puncture(s)
ONSF: Optic Nerve Sheath Fenestration
VA: Ventriculoatrial
VP: Ventriculoperitoneal
VPN: Virtual Private Network
